# Calcitriol increases MBNL1 expression and alleviates myotonic dystrophy phenotypes in HSA^LR^ mouse models

**DOI:** 10.1186/s12967-022-03806-9

**Published:** 2022-12-12

**Authors:** Kun Huang, Dan-Dan Wang, Wen-Bao Hu, Wei-Qian Zeng, Xia Xu, Qiu-Xiang Li, Fang-Fang Bi, Huan Yang, Jian Qiu

**Affiliations:** 1 Hunan Key Laboratory of Molecular Precision Medicine, Department of Neurology, Xiangya Hospital, Central South University, Changsha, Hunan China; 2 Department of Neurology, Xiangya Hospital, Central South University, Changsha, Hunan China; 3 Department of General Medicine, Xiangya Hospital, Central South University, Changsha, Hunan China; 4 Hunan Key Laboratory of Medical Genetics, School of Life Sciences, Central South University, Changsha, Hunan China; 5 National Clinical Research Center for Geriatric Disorders, Xiangya Hospital, Central South University, Changsha, Hunan China

**Keywords:** Myotonic dystrophy, MBNL1, Calcitriol, HSA^LR^ mouse, Neuromuscular disorders

## Abstract

**Background:**

Myotonic dystrophy type 1 (DM1), one of the most common forms of adult-onset muscular dystrophy, is caused by abnormally expanded CTG repeats in the 3′ untranslated region of the *DMPK* gene. The CUG repeats transcribed from the expanded CTG repeats sequestrate a splicing factor, MBNL1, causing the clinical symptoms in DM1. Nowadays, only symptomatic treatments are available for DM1, and no rational therapy is available. Recently, upregulation of MBNL1 expression has been found to be one of the promising therapies for DM1.

**Methods:**

All experiments were conducted in the C2C12 myoblasts and HSA^LR^ mice, a DM1 mouse model. Real-time PCR and western blot were used to detect the mRNA and protein level, respectively. The rotarod exercise, grip strength and hanging time were used to evaluate the muscle strength of mice.

**Results:**

In this study, we demonstrated that calcitriol, an active form of vitamin D3, increased MBNL1 in C2C12 mouse myoblasts as well as in HSA^LR^ mice model for DM1. In HSA^LR^ mice model, calcitriol improved muscle strength, and corrected aberrant splicing in skeletal muscle. Besides, calcitriol reduced the number of central nuclei, and improved muscle histopathology in HSA^LR^ mice. In addition, we identified that calcitriol upregulated MBNL1 expression via activating the promoter of *Mbnl1* in C2C12 myogenic cells.

**Conclusion:**

Our study suggests that calcitriol is a potential pharmacological strategy for DM1 that enhances MBNL1 expression.

**Supplementary Information:**

The online version contains supplementary material available at 10.1186/s12967-022-03806-9.

## Background

Myotonic dystrophy type 1 (DM1), also known as Steinert’s disease, is one of the most common forms of adult-onset muscular dystrophy, with a prevalence of 9.27 per 100,000 [1]. DM1 is caused by abnormal expansion of CTG repeats in the 3**’** untranslated region of the myotonic dystrophy protein kinase (DMPK) gene on chromosome 19 [2]. Unaffected individuals harbor 5–35 copies of CTG repeats, whereas DM1 patients carry 50 or more copies [3]. The CUG repeats transcribed from the abnormally expanded CTG repeats sequestrate muscleblind-like protein 1 (MBNL1), leading to the downregulation of free MBNL1 [4]. The limited MBNL1 availability leads to the aberrant regulation of alternative splicing of hundreds of genes, causing various clinical manifestations such as progressive muscle wasting, myotonia, insulin resistance, cardiac arrhythmia, cataracts, and intellectual deficits [5].

Recently, some therapeutic approaches have been designed for DM1 [6, 7]. There is numerous evidence that MBNL1 functions are the limiting factors in DM1, and the upregulation of MBNL1 expression is one of the promising therapies for DM1. In animal experiments, *Mbnl1* knockout mice recapitulate the main clinical symptoms for DM1, such as myotonia, aberrant splicing, cataracts, cardiac dysfunctions, and progressive skeletal muscle weakness [8, 9]. In complementary gain-of-function experiments, the overexpression of *Mbnl1* in skeletal muscle, using a recombinant adeno-associated viral vector in a murine model expressing 250 CTG repeats (HSA^LR^ mice), rescued myotonia, hyperexcitability, and aberrant splicing [5, 10]. MBNL1 upregulation in DM1 mice and DM1 *drosophila* is well tolerated without obvious side effects, and rescues several symptoms, such as myotonia, myopathy, and mis-splicing events [11, 12].

Vitamin D is a steroid hormone, of which approximately 80% is produced in the skin [13]. In the skin, 7-dehydrocholesterol is transferred into pre-vitamin D3 and then vitamin D3 under the action of ultraviolet-B radiation. Vitamin D3 is further converted to 25-hydroxyvitamin D [25(OH)D] in the liver and 1,25-hydroxyvitamin D (1,25(OH)2D, also called calcitriol), an active form of vitamin D3, in the kidney [14]. Previous studies found that DM1 patients have a reduction of circulating 25(OH)D, which correlates with the severity of the disease, suggesting that vitamin D supplement may alleviate some symptoms of DM1 patients [15, 16].

In this study, we first identified that calcitriol increases MBNL1 in C2C12 mouse myoblasts. Next, we confirmed that calcitriol improves muscle strength in HSA^LR^ mice model for DM1. Calcitriol enhances MBNL1 expression and corrects aberrant splicing in skeletal muscle of HSA^LR^ mice. Besides, calcitriol reduces the number of central nuclei, and improves muscle histopathology in HSA^LR^ mice. In addition, we identified that calcitriol upregulates MBNL1 expression via activating the promoter of *Mbnl1* in C2C12 myogenic cells. Our study suggests that calcitriol is a promising pharmacological strategy for DM1.

## Methods

### C2C12 cell culture and drug treatment

C2C12 myoblasts (National Collection of Authenticated Cell Cultures, serial: SCSP-505) were cultured in Dulbecco’s modified Eagle’s medium (DMEM, Gibco, Cat. C12430500BT) supplemented with 10% (v/v) fetal bovine serum (FBS, Procell, Cat. 164210-50) and incubated at 37 °C with 5% CO_2_. Different concentrations of calcitriol (MCE, HY-10,002) or ergocalciferol (Aladdin, E106318) were added into the culture medium of C2C12 myoblasts with 50–60% confluency for 24 h before analysis. To examine the effect of calcitriol or ergocalciferol in differentiated C2C12 myotubes, C2C12 myoblasts were grown to 95% confluency in DMEM with 10% FBS, and then changed into the differentiation medium containing 2% horse serum (HS) (Biological Industries, Cat. 04-004-1B) and various concentrations of calcitriol or ergocalciferol in DMEM for five days before analysis.

### Administration of calcitriol to DM1 mouse model

All mouse studies were approved by the Department of Laboratory Animals of Central South University, and were performed in accordance with the relevant guidelines. HSA^LR^ transgenic mice, which express approximately 250 untranslated CUG repeats driven by a muscle-specific promoter, were bought from The Jackson Laboratory, USA (Strain #:032031) [17]. The wild-type FVB/N (WT) mice that have the same genetic background were bought from the Department of Laboratory Animals of Central South University. Mice were divided into three groups: (1) untreated WT mice, (2) untreated HSA^LR^ mice, and (3) calcitriol treated HSA^LR^ mice. At 12 weeks after birth, mice started to take 0.9% normal saline or 1 µg/kg/day of calcitriol, which is a physiological dose widely used in mouse experiment [18, 19], by intraperitoneal injection for 12 weeks before sacrifice.

### Blood routine examination and vitamin D level analysis

After treated with or without calcitriol for 12 weeks, blood of the mice was collected from the angular veins and transferred into a blood collection tube containing EDTA to prevent blood clotting. Blood routine examination and cell classifications were analyzed using a Sysmex Hematology Analyzer (XN-1000-B1, Dewei, China) followed with manufacturer’s instructions. Vitamin D levels were determined using Mouse VD ELISA Kit from Shanghai ZCIBIO technology Co.,Ltd, (Cat. ZC-38,755), according to the manufacturer’s instructions.

### Rotarod test, grip of strength, and inverted mesh hanging test

For the rotarod test, the angular speed was accelerated from 0 to 40 rpm in 90 s with a 30 min maximum trail time (ZF44-YLS-4D, M407469). Mice were given two trials per week and the average endurance time of two trails for each animal was calculated. Mice with impaired muscle strength fell off quickly. The apparatus was cleaned between each trial to avoid odor interference.

The peak grip force was measured using a grip strength meter (YR92-YLS-13 A, M281330). Mice that grasp a wire mesh with the forelimbs and hindlimbs were pulled horizontally by their tails until they lost their grip. Measurements were performed twice and the average strength for each mouse was calculated.

Fatigability of limbs was tested for with the inverted grid hanging test [20]. Mice were placed on the center of an invertible 40×40 cm wire grid, mounted 60 cm above a padded surface. The time was recorded from the mice hang on to drop off. Each mouse was tested twice. The average hanging times were calculated for each mouse.

### Gene expression analysis and splicing analysis

RNA extraction from the tibialis anterior (TA) muscle of mouse was performed as described previously with minor modifications [21]. Tissues were mashed by High Speed Tissue Homogenizer (Servicebio, KZ-II) in the lysis buffer from the FastPure Cell/Tissue Total RNA Isolation Kit (Vazyme, Cat. RC101-01). Total RNA from the tissues and the cultured cells was extracted by the kit according to the manufacturer’s instructions. RNA was reverse-transcribed into cDNA using RevertAid Master Mix (Thermo Fisher, Cat. M1631).

Real-time RT-PCR was performed with the QuantStudio (Thermo Fisher Scientific) using the NovoStart® SYBR qPCR SuperMix Plus (Novoprotein, Cat. E096). Gene expression levels were normalized by that of glyceraldehyde-3-phosphate dehydrogenase (*Gapdh*). All real-time RT-PCR experiments were performed in duplicate. For splicing analysis, PCR amplifications were performed using 2×Taq Plus Master Mix II (Vazyme, Cat. P213) for 35 cycles. The primer sequences used for PCR are shown in Additional file 1: Table S1. The intensities of RT-PCR-amplified spliced products were quantified with the ImageJ program (http://imagej.nih.gov/ij/). We then estimated the ratio of exon inclusion by dividing the signal intensity of the upper band by the sum of signal intensities of two bands.

### Western blot and immunodecoration

Proteins from tissues and cultured cells were extracted by the lysis buffer [20 mM Tris/HCl pH 7.4, 50 mM NaCl, 10% (w/v) Glycerol, 0.1 mM EDTA, 2% SDS] supplemented with cOmplete protease inhibitor (Roche) and 1 mM PMSF. The protein concentration was quantified using Pierce 660 nm Protein Assay Reagent (Thermo Scientific, Cat. 23,225). Proteins were separated by SDS-PAGE, transferred onto polyvinylidene fluoride (PVDF) membranes (Immobilon-P, Millipore), and detected with primary and secondary antibodies listed in Additional file 1: Table S2 using ECL (Advansta, Cat. K-12,045-D50) and ChampChemi910 (Beijing Sage Creation Science Co., Ltd.).

### Histopathology of TA muscles

Pathological examinations were performed as described elsewhere with minor modifications [22–25]. Briefly, TA muscles of mouse were snap-frozen in isopentane chilled with liquid nitrogen. Quadriceps muscles were sliced at 8 μm with a cryostat. Hematoxylin and eosin (HE) staining was done according to the standard procedures. To determine the central nuclei, muscle fiber size and frequency distribution, tissue slides were observed using Olympus BX53 microscope. The cross-sectional area of the myofibers and the number of myonuclei was calculated from two random view per muscle sample using ImageJ.

### Transfection and luciferase assays

To make the luciferase vector harboring the *Mbnl1* promoter (pMbnl1), the mouse genomic region of chr3:60,407,525 − 60,408,852 according to GRCm37/mm9 was chemically synthesized, inserted into pGL3-Enhancer Vector (pGL3E, Promega) between BmtI and XhoI restriction sites, and was confirmed by Sanger sequencing (Sangon Biotech). Plasmids were introduced into Stbl3 Competent Cell (KTSM110L) and propagated. To measure the transcriptional activity of pMbnl1, C2C12 myoblasts were grown to 95% confluency in DMEM with 10% FBS, and then changed into the differentiation medium containing 2% HS and various concentrations of calcitriol. The cells were transiently transfected with pMbnl1 or pGL3E using Lipofectamine 3000 (ThermoFisher) according to the manufacturer’s recommendations. Five days later, the luciferase assay was performed using the Dual Luciferase Reporter Assay kit (Vazyme, Cat. DL101-01). For normalization, pRL-CMV Renilla Luciferase Reporter Vector was co-transfected, and the firefly luciferase activities were normalized to the *Renilla* luciferase activity.

## Results

### Calcitriol increases MBNL1 expression in myogenic C2C12 cells

**Fig. 1:**
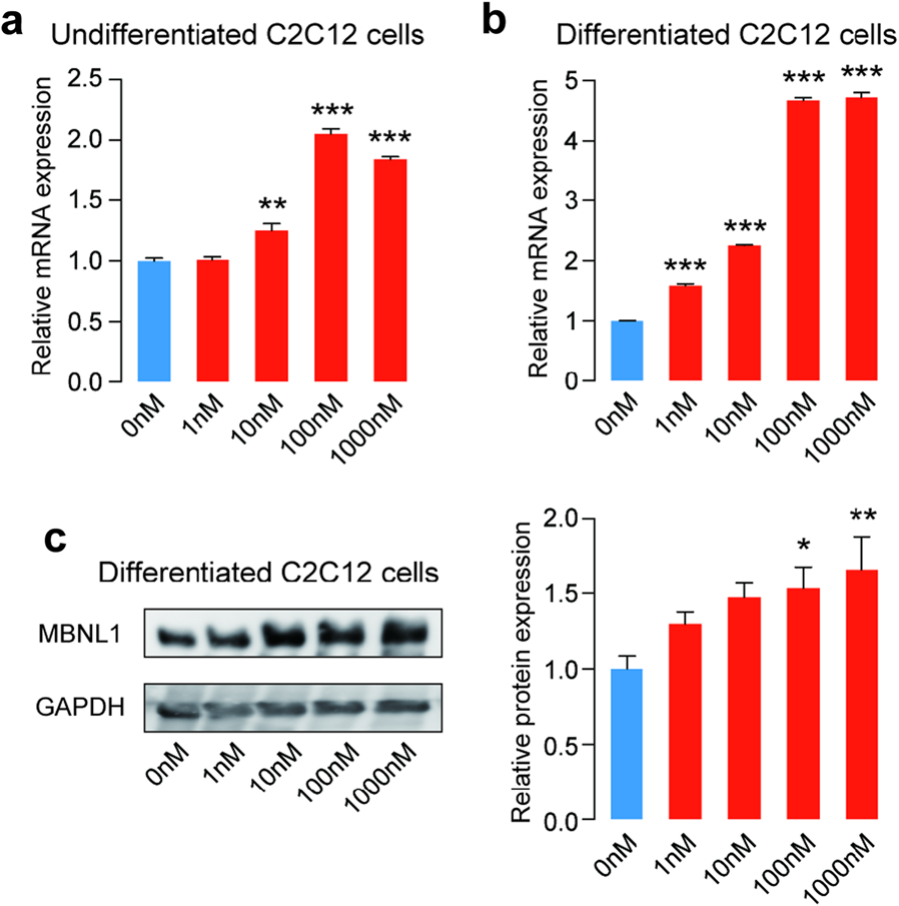
Calcitriol upregulates MBNL1 expression in C2C12 myoblasts and myotubes. Real-time RT-PCR analysis (**a**, **b**) and western blot (**c**) were performed using undifferentiated or differentiated C2C12 cells. The cells were treated with calcitriol as indicated. Undifferentiated C2C12 cells were examined one day after the treatment. Differentiated C2C12 cells were examined on day 5 of differentiation. Expression levels of *Mbnl1* mRNA and MBNL1 protein are normalized to those of *Gapdh* and GAPDH, respectively, and also to 0 nM-treated cells. Mean and SEM (*n* = 3 and 6 culture dishes for real-time RT-PCR and western blot, respectively) are indicated. *p < 0.05, **p < 0.01 and ***p < 0.001 by one-way ANOVA followed by Turkey multiple comparison correction

Since the upregulation of MBNL1 is a promising therapeutic target of DM1, and the level of circulating 25(OH)D (the precursor of calcitriol) correlates inversely with the disease severity in DM1 patients [15, 16], we started to address whether calcitriol regulates MBNL1 expression using C2C12 cells. We cultured the cells with different concentrations of calcitriol for 24 h and analyzed *Mbnl1* mRNA level by real-time RT-PCR. Calcitriol promoted the expression of *Mbnl1* mRNA up to two-fold in undifferentiated C2C12 cells (Fig. 1a). We next examined the effect of calcitriol on MBNL1 expression during myogenic differentiation. We harvested total RNA from C2C12 cells on differentiation day 5, and performed real-time RT-PCR analysis. Our analysis showed that calcitriol also upregulated *Mbnl1* mRNA in a dose-dependent manner in differentiated C2C12 cells (Fig. 1b). Consistently, western blot analysis showed that calcitriol upregulated MBNL1 protein level (Fig. 1c). Meanwhile, we did not observe any significant effect of calcitriol on the expression of muscle-related proteins, such as α-sarcoglycan [26] and Myogenin [27], in C2C12 cells (Additional file 1: Figure S1). Of note, ergocalciferol (an active form of vitamin D2) neither significantly affect *Mbnl1* expression (Additional file 1: Figure S2). These results suggest that calcitriol specifically enhances MBNL1 expression in myogenic cells in a dose-dependent manner.

### Calcitriol improves muscle strength in HSA^LR^ mice

**Fig. 2:**
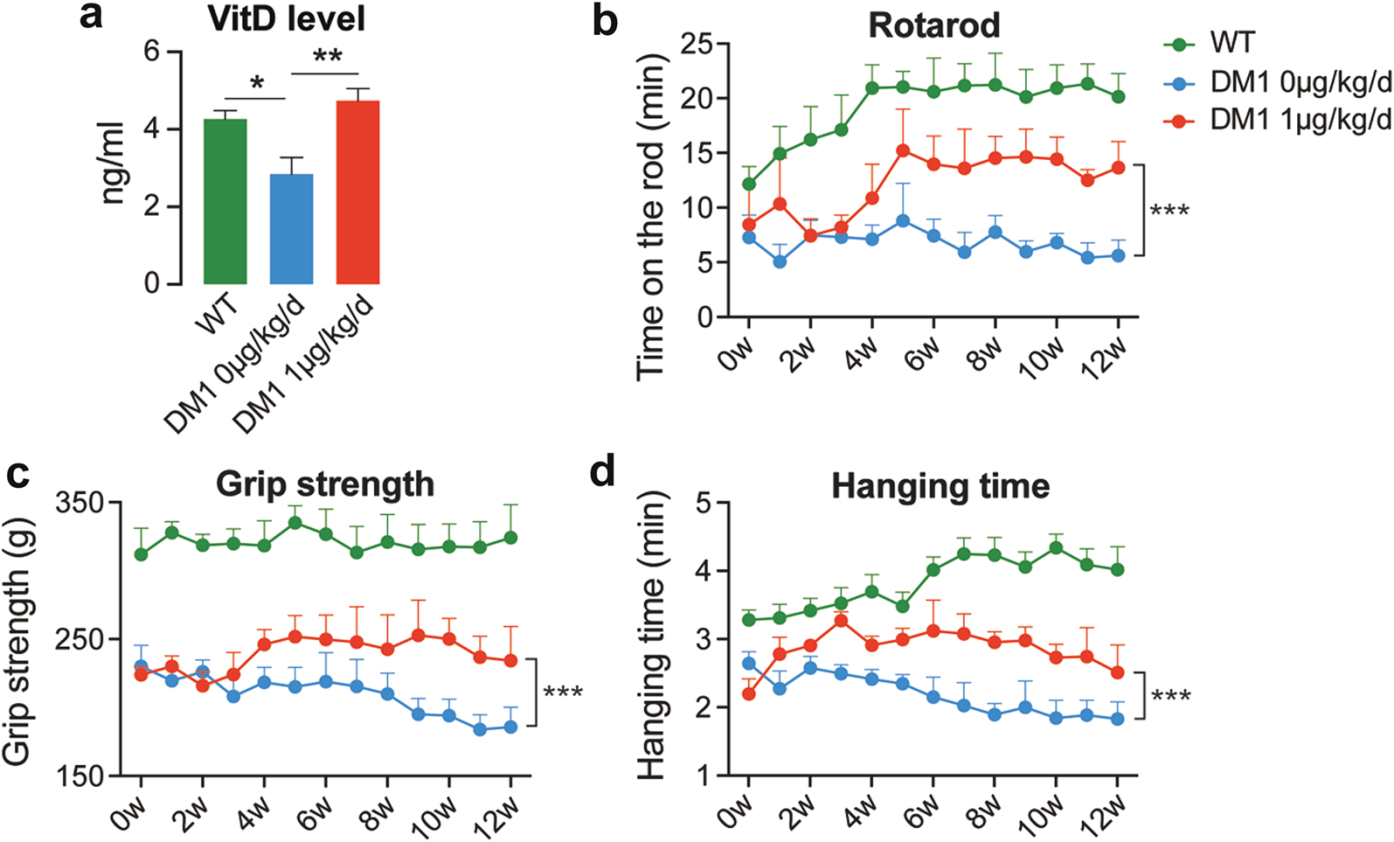
Calcitriol alleviates muscle weakness in HSA^LR^ mice. (**a**) WT and HSA^LR^ mice were treated with calcitriol as indicated. Vitamin D level of plasma from each group were analyzed. Mean and SEM (*n* = 8, 8 and 8 in WT, DM1 0 µg/kg/d and DM1 1 µg/kg/d, respectively) are indicated. (**b–d**) Calcitriol induced partial recovery from the muscle weakness occurring in the HSA^LR^ mice. The time that the mice stay on the rotarod (**b**), the grip strength (**c**) and the time that the mice hang on the wire mesh (**d**) of each group was recorded. Mean and SEM (*n* = 9, 9 and 8 in WT, DM1 0 µg/kg/d and DM1 1 µg/kg/d, respectively) are indicated. *p < 0.05, **p < 0.01 and ***p < 0.001 by one-way ANOVA followed by Turkey multiple comparison correction (**a**), and by two-way ANOVA followed by Turkey multiple comparison correction (**b–d**)

Next, we analyzed the effects of calcitriol on HSA^LR^ mice. HSA^LR^ mouse is a widely-used model for DM1, which carries 250 CUG repeats driven by a muscle-specific promoter. Calcitriol (1 µg/kg/day) was given to the HSA^LR^ mice by intraperitoneal injection for 12 weeks from age 12 weeks. We confirmed that the calcitriol treatment did not affect the body weight and blood cells composition in HSA^LR^ mice (Additional file 1: Figure S3). As expected, calcitriol upregulated vitamin D levels from 2.85 ng/ml (~ 6.8 nM) to 4.74 ng/ml (~ 11.4 nM) in HSA^LR^ mice (Fig. 2a). We next analyzed the rotarod exercise, grip strength and hanging time to evaluate the muscle strength of these mice during calcitriol treatment. We found that calcitriol treatment increased the exercising time on the rotarod, the grip strength and the hanging time on the wire mesh in HSA^LR^ mice (Fig. 2b–d). These results suggest that calcitriol ameliorates muscle weakness in DM1 mouse model.

### Calcitriol improves muscle histopathology in HSA^LR^ mice

**Fig. 3:**
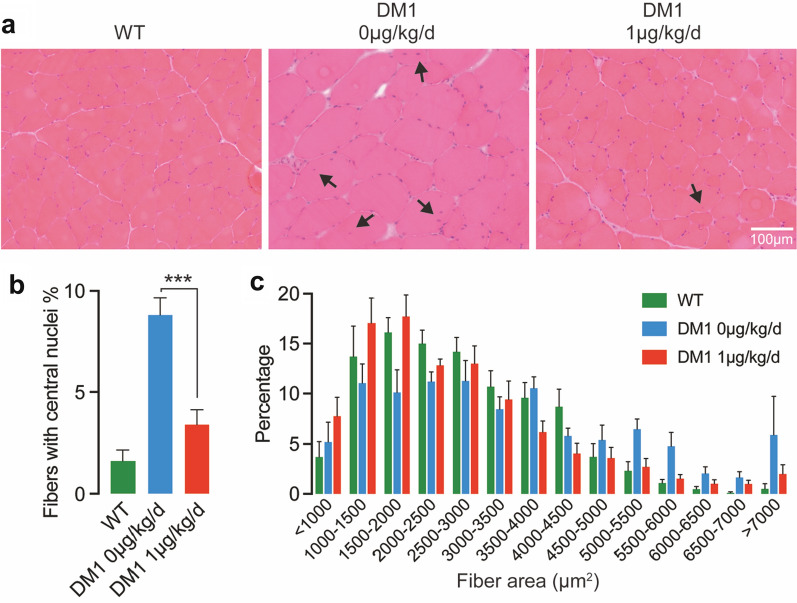
In HSA^LR^ mice, calcitriol improves muscle histopathology. (**a**) Representative H&E staining of TA muscles in different groups of mice as indicated. Arrows point to the central nuclei of muscle fibers. Scale bar = 100 μm. (**b**) The number of fibers with central nuclei was counted in three groups of mice. The data were analyzed by one-way ANOVA followed by Turkey multiple comparison correction. ***p < 0.001. (**c**) Frequency distribution of cross-sectional TA muscle fiber area was counted in three groups of mice. The mean and SEM (*n* = 8, 9 and 8 in WT, DM1 0 µg/kg/d and DM1 1 µg/kg/d, respectively) in each group are indicated

In HSA^LR^ mice, central nuclei and markedly increased fiber size variations are pathological features in skeletal muscles. We stained muscle sections of untreated wild type mice, untreated HSA^LR^ mice, and calcitriol-treated HSA^LR^ mice with hematoxylin and eosin (H&E), which could clearly show nuclei and muscle fiber size (Fig. 3a). We found that calcitriol significantly reduced the number of muscle fibers with central nuclei (Fig. 3b). The frequency distribution of the muscle fiber area further revealed that the TA muscle of calcitriol-treated HSA^LR^ mice had a shift toward WT mice as compared with the untreated HSA^LR^ mice (Fig. 3c).

### Calcitriol upregulates MBNL1 expression and corrects aberrant splicing in skeletal muscle

**Fig. 4:**
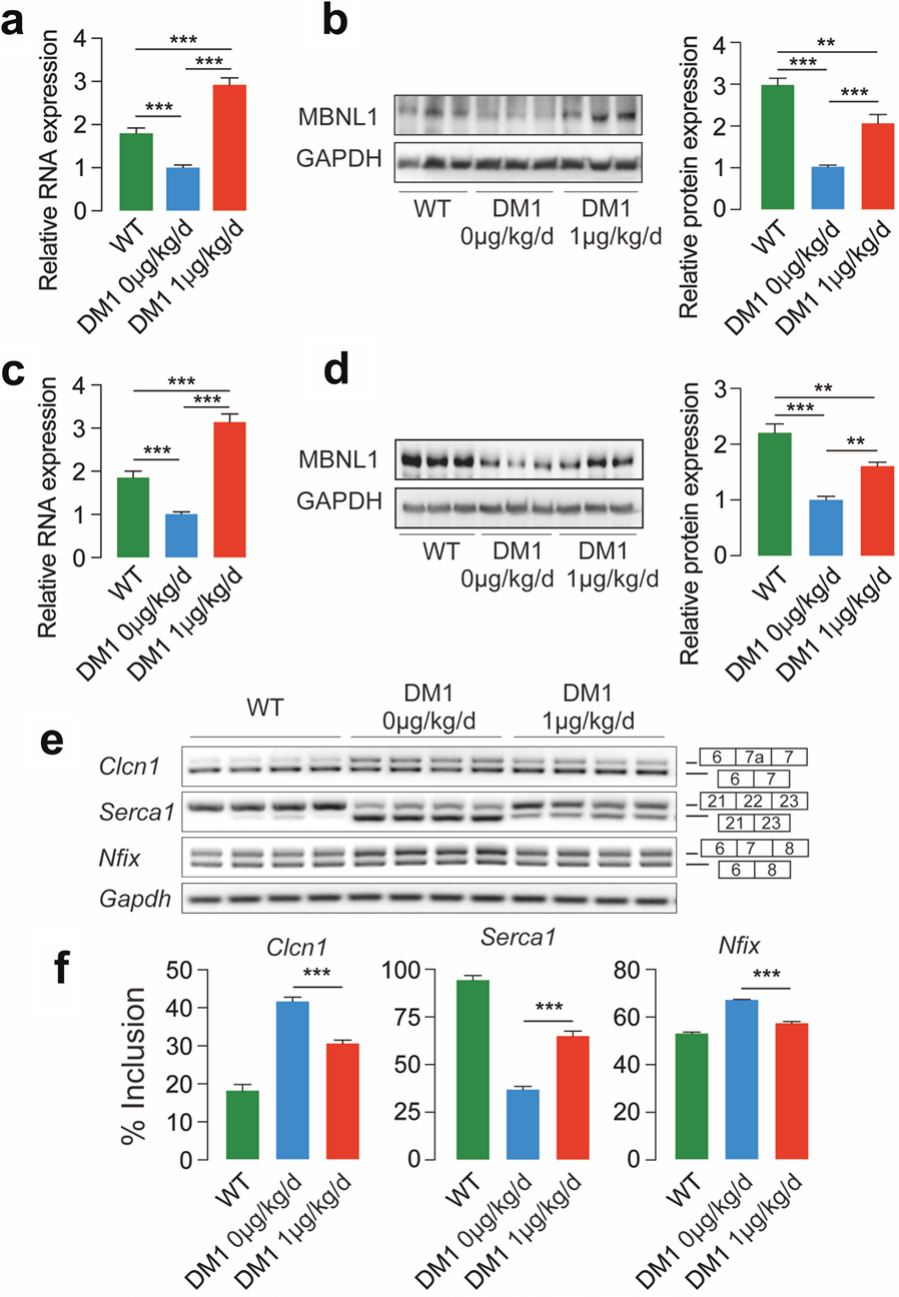
Calcitriol upregulates *Mbnl1* expression and corrects aberrant splicing in skeletal muscle of HSA^LR^ mice.(**a, c**) Real-time RT-PCR analysis to estimate the expression of *Mbnl1* in TA muscles (**a**) and soleus (**c**) of WT and HSA^LR^ mice treated with calcitriol as indicated. (**b, d**) Western blot analysis of MBNL1 in mouse TA muscles (**b**) and soleus (**d**). Left panel shows representative blots, and right panel shows the quantitative analysis of signal intensities. (**e**) RT-PCR analysis to evaluate the splicing of *Clcn1* exon 7a, *Serca1* exon 22 and *Nfix* exon 7 in TA muscles of four mice from each group as indicated. (**f**) The ratio of inclusion of relevant exon was calculated. The mean and SEM in each group are indicated. The data were analyzed by one-way ANOVA followed by Turkey multiple comparison correction. *p < 0.05, **p < 0.01 and ***p < 0.001

Consistent with the results of C2C12 cells, in vivo analysis showed that calcitriol elevated the expression of *Mbnl1* in TA muscles and soleus of HSA^LR^ mice (Fig. 4a–d). Calcitriol enhanced the *Mbnl1* mRNA level by approximate 3 folds and significantly elevated the amount of MBNL1 proteins. Interestingly, although the level of *Mbnl1* mRNA in calcitriol treated HSA^LR^ mice significantly exceeded the one in WT mice, the protein level was still significantly lower. Additional regulating mechanisms on translational/post-translational level of *Mbnl1* may be involved and await further studies. Since MBNL1 regulates splicing events in skeletal muscle, we assumed that the enhanced expression of MBNL1 by calcitriol may correct the aberrant splicing in HSA^LR^ mice. Indeed, RT-PCR analysis demonstrated that calcitriol significantly suppressed the abnormal inclusion of exon 7a in *Clcn1* in HSA^LR^ mice (Fig. 4e, f). Moreover, we also found that calcitriol ameliorated the aberrant splicing of *Serca1* exon 22 and *Nfix* exon 7 in HSA^LR^ mice (Fig. 4e, f). Thus, our results show that calcitriol enhances the MBNL1 expression and ameliorates the aberrant splicing in skeletal muscle in vivo.

### Calcitriol activates the promoter of ***Mbnl1*** in C2C12 myogenic cells

**Fig. 5:**
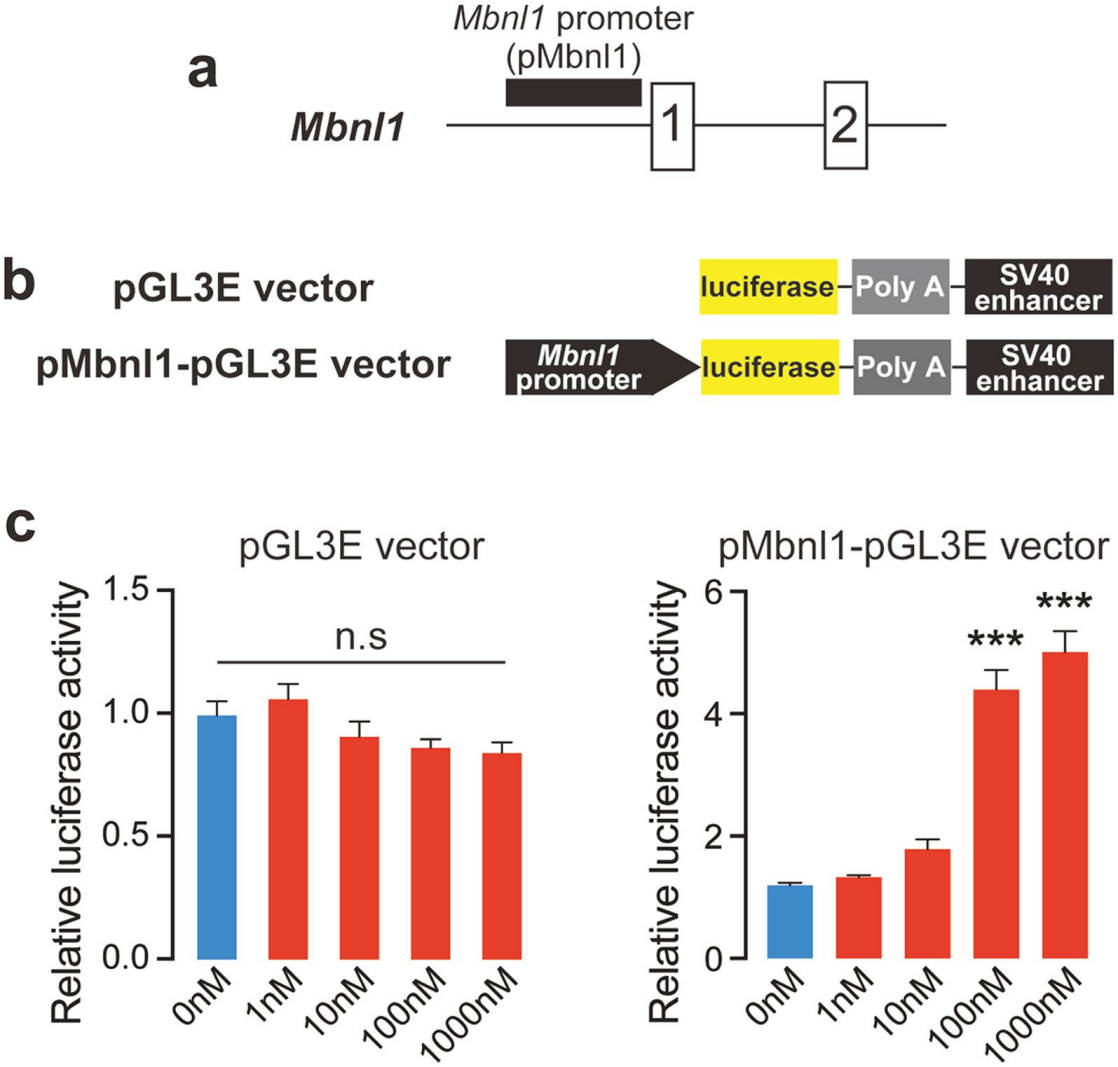
Calcitriol activates the promoter of ***Mbnl1*** in myogenic cells. (**a**) Schematic diagram showing the position of promoter of *Mbnl1* gene (pMbnl1). Exons are shown by boxes, introns by thin lines, and pMbnl1 by a black closed square. (**b**) Schematic diagram of pGL3E vector and pMbnl1-pGL3E vector. In pMbnl1-pGL3E, the pMbnl1 region was cloned into pGL3E upstream of the firefly luciferase gene. (**c**) Luciferase activity of pGL3E or pMbnl1-pGL3E during myogenic differentiation of C2C12 myoblasts. Cells were treated with different concentrations of calcitriol from differentiation day 0, whole cell lysates were extracted and firefly luciferase activity was evaluated on day 5 after induction of differentiation. Firefly luciferase activity was normalized to the *Renilla* luciferase activity of co-transfected pRL-CMV, and also to 0 nM treated cells. Mean and SD (*n* = 5 culture dishes) are indicated. ***p < 0.001, and n.s., not significant by one-way ANOVA followed by Turkey multiple comparison correction

A previous study identified the promoter of *Mbnl1* which locates in the 5’UTR (untranslated region) in mouse [28], we named pMbnl1 (Fig. 5a). To examine whether calcitriol promotes the activity of pMbnl1, we inserted the promoter sequence upstream of the firefly luciferase cDNA to make pMbnl1-pGL3E (Fig. 5b). C2C12 myoblasts were introduced with pGL3E or pMbnl1-pGL3E, and were induced to differentiate 24 h later. In order to examine the effect of calcitriol on the promoter activity, cells were treated with various concentrations of calcitriol during myogenic differentiation. Luciferase assay revealed that calcitriol significantly increased the luciferase activity of pMbnl1-pGL3E, not the control plasmid (Fig. 5c). Thus, calcitriol could enhance the activity of *Mbnl1* promoter to upregulate the MBNL1 protein level.

## Discussion

DM1 is a rare, multisystem disorder without curative or disease-modifying treatment to slow or stop disease progression [29]. Symptomatic and supportive treatment, preventive measures and clinical surveillance are the currently available options for DM1 patients [30]. The upregulation of MBNL1 protein level is a promising therapeutic strategy against DM1. Previous studies indicated that MBNL1 is downregulated in DM1 models and patients, and the overexpression of MBNL1 is beneficial in murine disease models [5, 12]. Various drugs and epigenetic approaches have been found to promote endogenous MBNL1 expression and ameliorate the disease phenotypes in different experimental models [31–34]. In the current study, we identified that calcitriol (active form of vitamin D3) elevated MBNL1 expression in mouse myogenic cells and in skeletal muscles of HSA^LR^ mouse model via enhancing the *Mbnl1* promoter activity.

Vitamin D plays essential roles in skeletal muscle. Vitamin D positively influences the protein synthesis and increases the size of muscle cells, leading to increased muscle mass, strength and performance [35, 36]. Meta-analyses also reported beneficial effects of vitamin D supplementation on muscle strength and function in healthy adults [37, 38]. Accordingly, vitamin D deficiency leads to muscle weakness and muscle mass reduction [39, 40]. Clinical studies also identified a correlation between serum 25(OH)D concentration and muscle strength and function in older adults [41, 42]. Importantly, vitamin D deficiency has been found to correlate with the severity of the disease symptoms in DM1 patients [15, 16]. In our study, the upregulation of MBNL1 by calcitriol seems to be beneficial in the DM1 model, since the abnormal splicing of MBNL1-regulated genes (*Clcn1*, *Serca1*, and *Nfix*) was partially corrected, and the muscle pathology as well as the muscle weakness was ameliorated. These results suggest that calcitriol upregulates MBNL1 expression and improves muscle strength in HSA^LR^ mice model.

MBNL1 is a splicing factor which can promote opposite splicing patterns for different genes depending on pre-mRNA binding context and interacting proteins [8, 43]. The strength of alternative exon exclusion or inclusion highly depends on the MBNL1 binding affinity, relative concentration and local RNA structure features [44]. MBNL1 could bind to the alternative exon or the upstream intron, causing exon skipping. It could also bind to the end of the alternative exon or to the downstream intron, promoting exon inclusion. This regulated alternative splicing activity of MBNL1 could potentially explain the observed differential effects of elevated MBNL1 on the aberrant splicing of *Clcn1*, *Nfix* and *Serca1* in calcitriol treated HSA^LR^ mice.

MBNL1 sequestration by abnormally expanded CUG repeats is key to the clinical symptoms in DM1 [8], but this multi-systemic disease has also been found to be related with many other factors, such as increased CELF [45] or HNRNPA1 [46] expression, and decreased DMPK, SIX5, and DMWD expression [47]. In line with this notion, although calcitriol could significantly upregulate MBNL1 protein level in the DM1 mouse model to ~ 70% of WT, it could only partially rescue various phenotypes of the disease model. Therefore, it would be interesting in the future to search for more potent chemicals, with structures similar to calcitriol or not, with therapeutic potential for DM1.

The involvement of calcitriol in the regulation of promoter activity has been previously reported. In target tissues, calcitriol binds to vitamin D receptor (VDR) and retinoid X receptor (RXR) [48, 49]. The calcitriol-VDR-RXR complex binds to vitamin D response element (VDRE) in the promoter region of calcitriol-target genes [50]. This binding leads to the recruitment of enzymatic coregulatory complexes that shift the local chromatin structure, facilitate the epigenetic modification of histones and improve the local concentration of RNA polymerase II (RNA pol II), leading to the regulation of mRNA expression [50]. In our study, we found that calcitriol activates *Mbnl1* promoter and increases MBNL1 expression. However, the exact molecular mechanisms still remain unclear. The multiple predicted MEF2 binding sites (http://jaspar.genereg.net/) in the *Mbnl1* promoter region may be involved and awaits further studies [51].

## Conclusion

Taken together, we reported that calcitriol activates *Mbnl1* promoter, increases the endogenous MBNL1 protein expression, and improves muscle strength and function in DM1 mouse model, suggesting calcitriol as a potential pharmacological option for DM1 patients.

## Supplementary Information

**Additional file 1: Figure S1. **Calcitriol has no significanteffect on the expression of α-sarcoglycan and Myogenin in C2C12 myoblasts andmyotubes. Real-timeRT-PCR analysis (a,b) and westernblot (c) were performed usingundifferentiated or differentiated C2C12 cells. The cells were treated withcalcitriol asindicated.Undifferentiated C2C12 cells were examined one day after the treatment.Differentiated C2C12 cells were examined on day 5 of differentiation.Expression levels of *α-sarcoglycan *(a) and* Myogenin *mRNA (b) and α-sarcoglycan and Myogenin protein (c) are normalized to those of *Gapdh* and GAPDH, respectively, and also to 0 nM-treated cells. Mean and SEM (*n* = 3 and 4 culture dishes for real-time RT-PCR and western blot, respectively) areindicated. n.s,not significant byone-way ANOVA followed by Turkey multiple comparison correction. **Figure S2.** Vitamin D2 has nosignificant effect on expression of *Mbnl1*in C2C12 myoblasts and myotubes. Real-time RT-PCR analysis were performed using undifferentiated (a) or differentiated C2C12 cells (b). The cells were treated with vitamin D2 as indicated. Undifferentiated C2C12 cellswere examined one day after the treatment. Differentiated C2C12 cells wereexamined on day 5 of differentiation. Expression levels of *Mbnl1* are normalized to those of *Gapdh*, respectively, and also to 0 nM-treated cells. Mean and SEM (*n* = 3 culture dishes) are indicated. n.s, not significant by one-way ANOVA followed byTurkey multiple comparison correction. **Figure S3.** In HSA^LR^mice, calcitriol has no effect on body weight, blood cell number, and hemoglobin.Threegroups of mice were analyzed: (i) Untreated wildtype FVB/N mice (WT); (ii)untreated HSA^LR^ mice (DM1 0μg/kg/d); and (iii) 1μg/kg/d calcitriol-treatedHSA^LR^ mice (DM1 1μg/kg/d). (a)Changes of body weight after treated with or without calcitriol in each group. Mean and SEM (*n* = 9, 9 and 8 in WT, DM1 0μg/kg/d and DM1 1μg/kg/d,respectively) areindicated. (b-e) Number of red blood cell (RBC),white blood cell (WBC), and platelets (PLT), and content of hemoglobin (HFB) ineach group treated with or without calcitriol. Mean and SEM (*n*= 8, 8 and 8 in WT, DM10μg/kg/d and DM1 1μg/kg/d, respectively) are indicated. *p < 0.05, **p <0.01, ***p < 0.001 and n.s, not significant by two-way ANOVA followed by Turkeymultiple comparison correction (a),and by one-way ANOVA followed by Turkey multiple comparison correction (b-e). **Figure S4.** Full range imagesof the cropped gels.Full range images of the cropped gels presented in Figure 1c (a), Figure 4b (b) and Figure 4d (c). **Figure S5.** Full rangeimages of the cropped gels presented in Supplementary Figure S1c. **Table S1. **Primer sequences for PCR. **Table S2. **Antibodies and dilutions for Western blot.

## Data Availability

The data presented in this study are available on request from the corresponding author.

## Abbreviations

DM1: Myotonic dystrophy type 1
DMPK: Myotonic dystrophy protein kinase
MBNL1: Muscleblind-like protein 1
25(OH)D: 25-hydroxyvitamin D
1,25(OH)2D: 1,25-hydroxyvitamin D
DMEM: Dulbecco’s modified Eagle’s medium
HS: Horse serum
WT: Wild-type FVB/N mice
TA: Tibialis anterior
PVDF: Polyvinylidene fluoride
HE: Hematoxylin and eosin
pMbnl1: *Mbnl1* promoter
VDR: Vitamin D receptor
RXR: Retinoid X receptor
VDRE: Vitamin D response element
RNA pol II: RNA polymerase II
