# Supplementary material for: Calcitriol increases MBNL1 expression and alleviates myotonic dystrophy phenotypes in HSALR mouse models
Source: J Transl Med. 2022 Dec 12;20:588. doi: 10.1186/s12967-022-03806-9 (PMC9743610; doi:10.1186/s12967-022-03806-9)
Supplement: Supplementary file 1 — Additional file 1: Figure S1. Calcitriol has no significanteffect on the expression of α-sarcoglycan and Myogenin in C2C12 myoblasts andmyotubes. Real-timeRT-PCR analysis (a,b) and westernblot (c) were performed usingundifferentiated or differentiated C2C12 cells. The cells were treated withcalcitriol asindicated.Undifferentiated C2C12 cells were examined one day after the treatment.Differentiated C2C12 cells were examined on day 5 of differentiation.Expression levels of α-sarcoglycan (a) and Myogenin mRNA (b) and α-sarcoglycan and Myogenin protein (c) are normalized to those of Gapdh and GAPDH, respectively, and also to 0 nM-treated cells. Mean and SEM (n = 3 and 4 culture dishes for real-time RT-PCR and western blot, respectively) areindicated. n.s,not significant byone-way ANOVA followed by Turkey multiple comparison correction. Figure S2. Vitamin D2 has nosignificant effect on expression of Mbnl1in C2C12 myoblasts and myotubes. Real-time RT-PCR analysis were performed using undifferentiated (a) or differentiated C2C12 cells (b). The cells were treated with vitamin D2 as indicated. Undifferentiated C2C12 cellswere examined one day after the treatment. Differentiated C2C12 cells wereexamined on day 5 of differentiation. Expression levels of Mbnl1 are normalized to those of Gapdh, respectively, and also to 0 nM-treated cells. Mean and SEM (n = 3 culture dishes) are indicated. n.s, not significant by one-way ANOVA followed byTurkey multiple comparison correction. Figure S3. In HSALRmice, calcitriol has no effect on body weight, blood cell number, and hemoglobin.Threegroups of mice were analyzed: (i) Untreated wildtype FVB/N mice (WT); (ii)untreated HSALR mice (DM1 0μg/kg/d); and (iii) 1μg/kg/d calcitriol-treatedHSALR mice (DM1 1μg/kg/d). (a)Changes of body weight after treated with or without calcitriol in each group. Mean and SEM (n = 9, 9 and 8 in WT, DM1 0μg/kg/d and DM1 1μg/kg/d,respectively) areindicated. (b-e) Number of red blood cell (RBC),white blood cell (W [file 12967_2022_3806_MOESM1_ESM.docx]

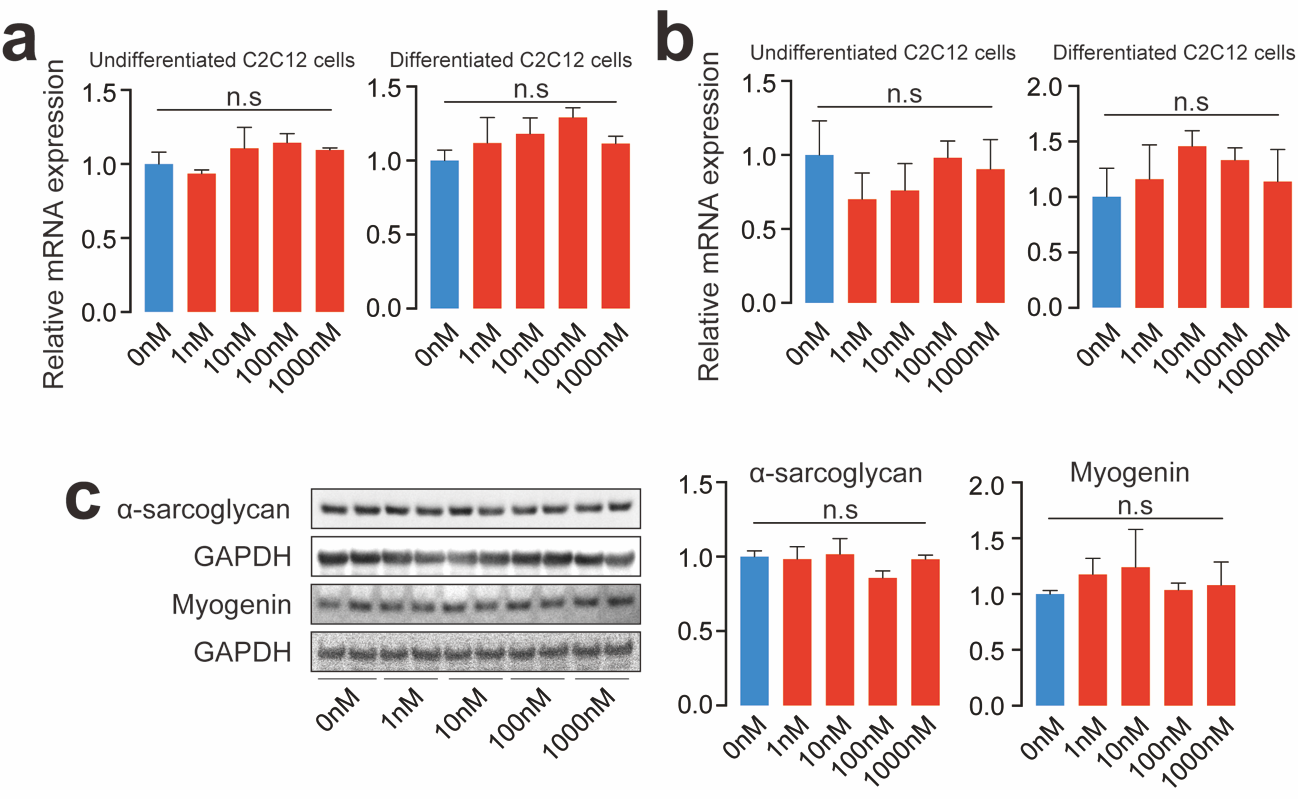


**Supplementary Figure S1. Calcitriol has no significant effect on the expression of α-sarcoglycan and Myogenin in C2C12 myoblasts and myotubes.** Real-time RT-PCR analysis **(a,b)** and western blot **(c)** were performed using undifferentiated or differentiated C2C12 cells. The cells were treated with calcitriol as indicated. Undifferentiated C2C12 cells were examined one day after the treatment. Differentiated C2C12 cells were examined on day 5 of differentiation. Expression levels of *α-sarcoglycan* **(a)** and *Myogenin* mRNA **(b)** and α-sarcoglycan and Myogenin protein **(c)** are normalized to those of *Gapdh* and GAPDH, respectively, and also to 0 nM-treated cells. Mean and SEM (*n* = 3 and 4 culture dishes for real-time RT-PCR and western blot, respectively) are indicated. n.s, not significant by one-way ANOVA followed by Turkey multiple comparison correction.


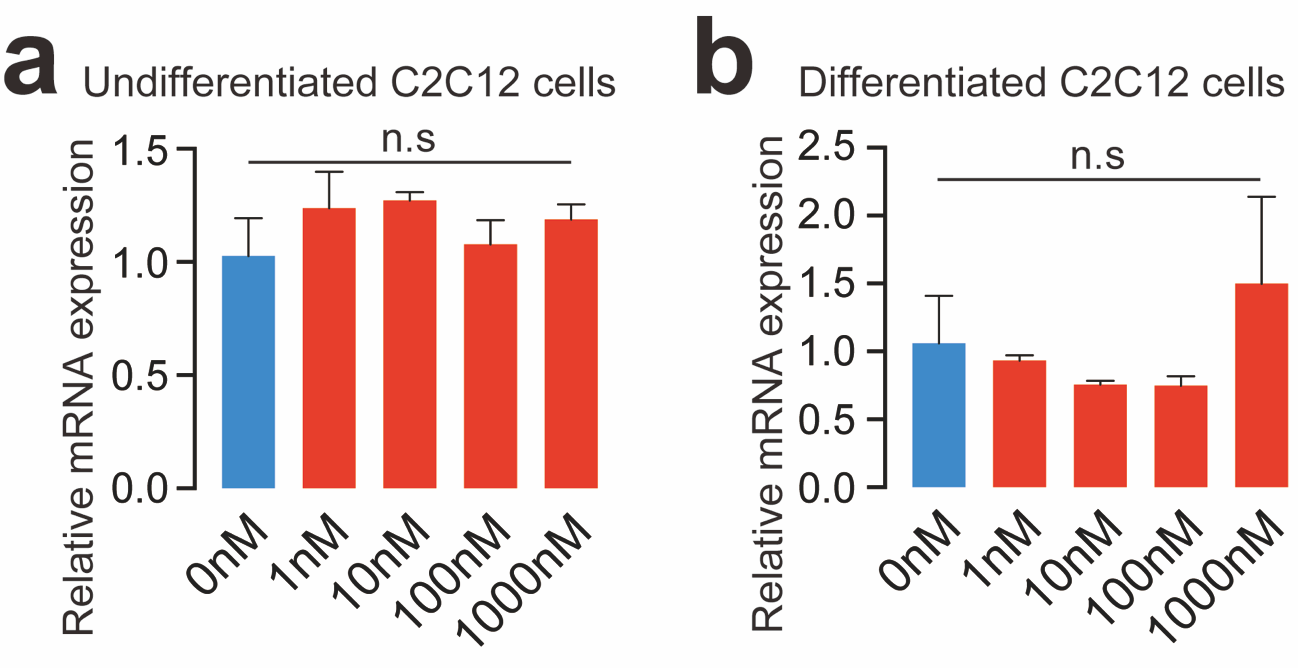


**Supplementary Figure S2. Vitamin D2 has no significant effect on expression of *Mbnl1* in C2C12 myoblasts and myotubes.** Real-time RT-PCR analysis were performed using undifferentiated (**a**) or differentiated C2C12 cells (**b**). The cells were treated with vitamin D2 as indicated. Undifferentiated C2C12 cells were examined one day after the treatment. Differentiated C2C12 cells were examined on day 5 of differentiation. Expression levels of *Mbnl1* are normalized to those of *Gapdh*, respectively, and also to 0 nM-treated cells. Mean and SEM (*n* = 3 culture dishes) are indicated. n.s, not significant by one-way ANOVA followed by Turkey multiple comparison correction.


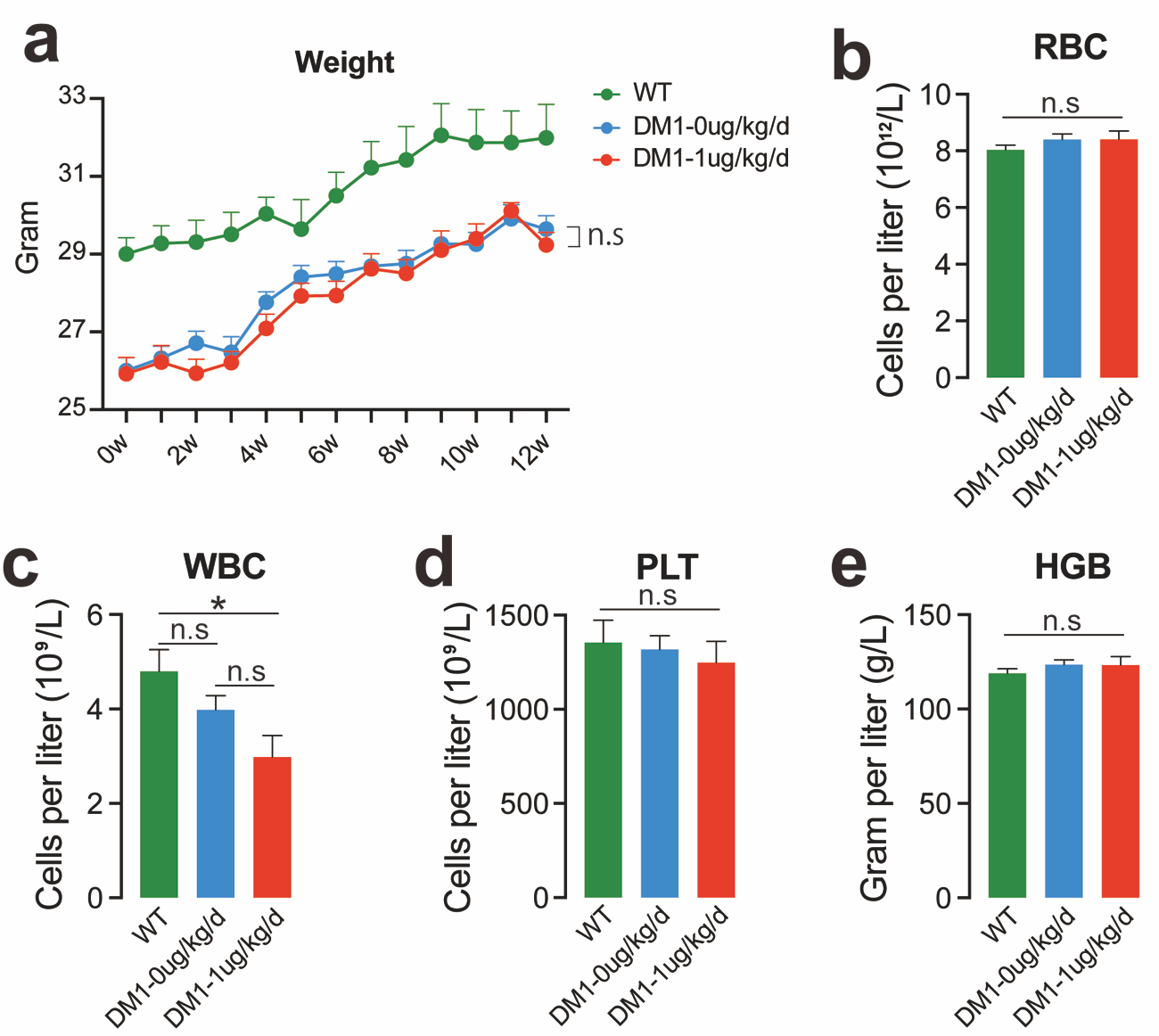


**Supplementary Figure S3. In HSA^LR^ mice, calcitriol has no effect on body weight, blood cell number, and hemoglobin.** Three groups of mice were analyzed: (i) Untreated wildtype FVB/N mice (WT); (ii) untreated HSA^LR^ mice (DM1 0μg/kg/d); and (iii) 1μg/kg/d calcitriol-treated HSA^LR^ mice (DM1 1μg/kg/d). **(a)** Changes of body weight after treated with or without calcitriol in each group. Mean and SEM (*n* = 9, 9 and 8 in WT, DM1 0μg/kg/d and DM1 1μg/kg/d, respectively) are indicated. **(b-e)** Number of red blood cell (RBC), white blood cell (WBC), and platelets (PLT), and content of hemoglobin (HFB) in each group treated with or without calcitriol. Mean and SEM (*n* = 8, 8 and 8 in WT, DM1 0μg/kg/d and DM1 1μg/kg/d, respectively) are indicated. **p* < 0.05, ***p* < 0.01, ****p* < 0.001 and n.s, not significant by two-way ANOVA followed by Turkey multiple comparison correction **(a)**, and by one-way ANOVA followed by Turkey multiple comparison correction **(b-e)**.


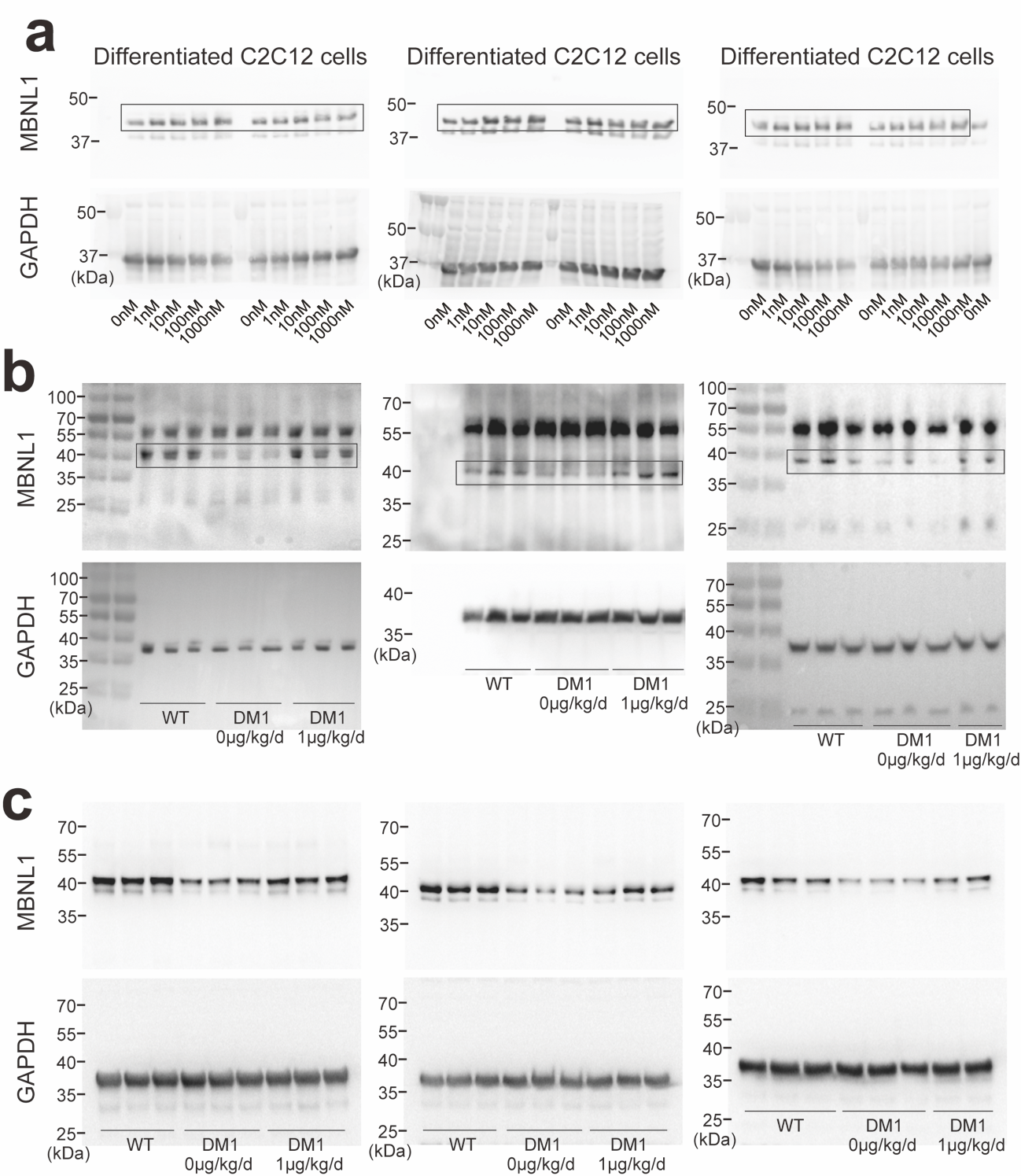


**Supplementary Figure S4. Full range images of the cropped gels.** Full range images of the cropped gels presented in Figure 1c **(a)**, Figure 4b **(b)** and Figure 4d **(c)**.


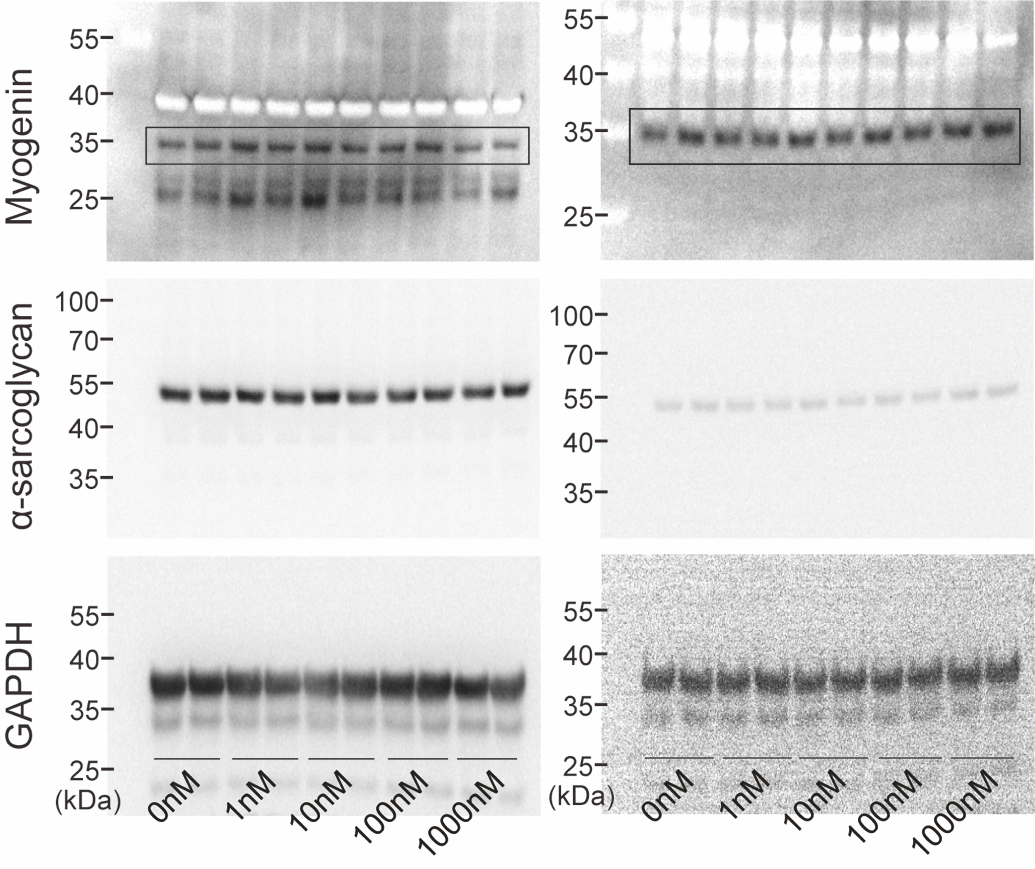


**Supplementary Figure S5. Full range images of the cropped gels presented in Supplementary Figure S1c.**

**Supplementary Table S1. Primer sequences for PCR**

| **Gene** | **Forward primer (5'-3')** | **Reverse primer (5'-3')** |
| --- | --- | --- |
| *Gapdh* | ACCCCTTCATTGACCTCAAC | TCCCGTTGATGACAAGCTTC |
| *Mbnl1* | GCGTGGCAATTGCAACAG | GCATCTCCCCTTGATGTAATCC |
| *α-sarcoglycan* | ACTTCCGCGTTGACTGGTGCAA | CACCAAGGCATCTGTCAGGAAG |
| *Myogenin* | CCATCCAGTACATTGAGCGCCT | CTGTGGGAGTTGCATTCACTGG |
| *Clcn1* | GTCCTCAGCAAGTTTATGTCC | GAATCCTCGCCAGTAATTCC |
| *Serca1* | ATCTTCAAGCTCCGGGCCCT | CAGCTTTGGCTGAAGATGCA |
| *Nfix* | TCGACGACAGTGAGATGGAG | CAAACTCCTTCAGCGAGTCC |

**Supplementary Table S2. Antibodies and dilutions for Western blot**

|  | Manufacturer | Species | Catalog No. | Dilution |
| --- | --- | --- | --- | --- |
| Primary antibodies |  |  |  |  |
| anti-MBNL1 | Santa Cruz | Mouse | sc-47740 | 1:200 |
| anti-GAPDH | ImmunoWay | Rabbit | YN5585 | 1:5000 |
| anti-α-sarcoglycan | Santa Cruz | Mouse | sc-271321 | 1:500 |
| anti-Myogenin | Santa Cruz | Mouse | sc-12732 | 1:500 |
| Second antibodies |  |  |  |  |
| HRP-conjugated anti-mouse IgG | Jackson ImmunoResearch | Goat | 115-035-146 | 1:3000 |
| HRP-conjugated anti-rabbit IgG | Sangon Biotech | Goat | D110058 | 1:5000 |
